# Respiration Rate Associations with Temperature and Relative Humidity and Preliminary Model-Derived Reference Points in Korean Native Black Goats

**DOI:** 10.3390/ani16203297

**Published:** 2026-10-20

**Authors:** Wonseok Cho, Woochurl Jeong, Hochan Kang, Wongi Min, Beomjin Park, Sangwoo Park, Honghee Chang

**Affiliations:** 1 Department of Animal Science, Gyeongsang National University, Jinju 52828, Republic of Korea; 2 Livestock Farm, Gyeongsang National University, Jinju 52635, Republic of Korea; 3 College of Veterinary Medicine, Gyeongsang National University, Jinju 52828, Republic of Korea; 4 Department of Information and Statistics, Gyeongsang National University, Jinju 52828, Republic of Korea; 5 Division of Animal Bioscience and Integrated Biotechnology, Gyeongsang National University, Jinju 52828, Republic of Korea; 6 Institute of Agriculture and Life Science, Gyeongsang National University, Jinju 52828, Republic of Korea

**Keywords:** environmental monitoring, relative humidity, small ruminants, thermal environment, thermoregulation

## Abstract

Quantitative evidence on the associations of temperature, relative humidity, and skin temperature (ST) with respiration rate (RR) in Korean native black goats remains limited, including potential sex- and growth-stage differences. Twenty-four goats (six per sex-by-growth-stage group) were repeatedly measured on 35 dates at a single farm over 15–33 °C, yielding 840 repeated animal-date observations. Natural-log-transformed RR was analyzed using a linear mixed-effects model accounting for animal-, date-, and pen-level dependence. Within the observed conditions, each 1 °C increase in temperature and 1 percentage-point increase in relative humidity were associated with 9.23% and 0.79% increases in RR, respectively. At 24 °C, mean relative humidity, and mean baseline body weight, adult goats had higher predicted RR than growing goats, while temperature-associated RR slopes did not clearly differ by sex or growth stage. Adding ST modestly improved in-sample model fit. Model-derived temperatures corresponding to RR criteria of 40, 60, and 80 breaths/min were 21.2–23.1, 25.8–27.7, and 29.0–31.0 °C, respectively, conditional on mean relative humidity and mean baseline body weight. These preliminary population-level reference points are not validated thresholds of heat-stress severity, welfare impairment, or production loss and require external validation in larger, multi-farm studies using independent physiological, behavioural, welfare, and production outcomes.

## Simple Summary

Heat stress can impair livestock health and productivity, but quantitative information on thermal responses of Korean native black goats remains limited. We repeatedly measured 24 goats at a single farm over a temperature range of 15–33 °C to examine associations of temperature and relative humidity with respiration rate and assess the additional information provided by skin temperature. Higher respiration rates were associated with higher temperature and relative humidity. At 24 °C and the mean relative humidity, adult goats had a higher predicted respiration rate than growing goats after adjustment for baseline body weight. Temperature-related changes in respiration rate did not clearly differ by sex or growth stage. Adding skin temperature modestly improved the model’s explanation of respiration rate in this sample. Model-derived temperatures corresponding to respiration rates of 40, 60, and 80 breaths/min ranged from approximately 21.2 to 23.1, 25.8 to 27.7, and 29.0 to 31.0 °C, respectively, at mean relative humidity and mean baseline body weight. These estimates are preliminary population-level reference points and should not be interpreted as validated thresholds of heat-stress severity, welfare impairment, or production loss. External validation is needed before wider application.

## 1. Introduction

The frequency and intensity of extreme heat events have increased in recent years due to climate change, and these changes have been identified as key environmental drivers that increase the risk of heat exposure in livestock production systems [1,2]. Under hot conditions, livestock exhibit physiological responses such as increased respiration rate (RR), elevated body temperature, and altered water metabolism to maintain thermal homeostasis; however, these responses may be accompanied by reduced feed intake and decreased productivity [3,4,5]. Panting is a major pathway of respiratory evaporative heat loss in mammals and is characterized by an increase in respiratory frequency accompanied by a decrease in tidal volume, thereby maximizing evaporative cooling in the upper airways [6,7]. In ruminants, panting is also regarded as an important heat-dissipation mechanism under increased heat load [4,8]. Therefore, RR can be used as a practical physiological indicator that rapidly reflects heat-related responses under field conditions [2,9]. Because RR can be assessed repeatedly and non-invasively under farm conditions and is directly related to respiratory evaporative heat dissipation, it is particularly suitable for deriving preliminary operational reference points for thermal monitoring in Korean native black goats.

In Korean livestock production, black goat production has expanded with increasing demand for functional health foods and medicinal products [10,11]. According to a recent national goat-industry report [10], the number of goat farms in Korea was 10,263 in 2023, with approximately 420,000 goats raised nationwide, making goats the second-largest livestock sector after Hanwoo cattle in terms of the number of farms. Goats are generally regarded as small ruminants with relatively high adaptability to diverse climatic conditions [12]. Recent studies in other goat breeds have likewise reported increases in RR and other physiological responses under elevated thermal conditions, including changes in surface or rectal temperature, feed or water intake, and metabolic responses [13,14,15]. However, the magnitude and pattern of heat-stress responses may differ according to breed or genetic background, sex, growth stage, and housing environment [9,16]. Sex and growth stage were therefore included as key biological factors to determine whether RR responses to thermal conditions differed among major animal groups within this breed. In dairy goats, heat stress has repeatedly been reported to increase RR and temperature-related physiological responses while negatively affecting feed intake and lactational performance [17,18]. However, these findings may not be directly generalizable to Korean native black goats because thermal responses can vary according to breed, genetic background, and production conditions. Korean native black goats have distinct production and management characteristics [11], and physiological data on their heat-stress responses are needed to develop preliminary population-level reference information and evaluate possible differences according to sex and growth stage. Such information is also necessary for developing housing and management strategies aimed at mitigating heat stress in goats [17,19].

Heat-related conditions in livestock are often evaluated using integrated environmental indices such as the temperature–humidity index (THI) [20]. However, the most appropriate THI model and corresponding heat-stress thresholds may vary according to regional climatic conditions and breed characteristics [20,21]. For the present objective, temperature (T) and relative humidity (RH) were evaluated separately rather than combined into a predefined THI because the study aimed to quantify their respective associations with RR and to develop prediction equations based on directly measured environmental variables. This approach also avoided imposing a THI formulation or threshold that has not been specifically validated for Korean native black goats. In addition to environmental conditions, animal-based thermal responses may provide complementary information. Infrared thermography-based skin temperature (ST) has been proposed as a useful noninvasive indicator of the thermal status of livestock [22]. Because RR reflects respiratory evaporative heat dissipation [4,6,7,8], ST was evaluated alongside RR to determine whether it provided additional information after adjustment for environmental and animal-related factors.

Nevertheless, although Korean native black goats are an indigenous goat breed of Korea and previous studies have addressed their production and management characteristics [11], quantitative evidence remains limited regarding the associations of T and RH with RR in this breed, whether temperature-associated RR responses differ according to sex and growth stage, and whether ST provides additional information after adjustment for environmental and animal-related factors. In particular, repeated observations from the same animals, environmental conditions shared by animals measured on the same date, and housing conditions shared by animals within the same pen require an analytical framework that accounts for the resulting dependence among observations. Moreover, possible differences in temperature responses according to sex and growth stage should be evaluated using a single integrated model rather than separate regression models for each group. Because summer conditions in Korea frequently involve both high temperature and high relative humidity [23,24], evaluating these relationships under field thermal conditions is particularly relevant.

Accordingly, the following hypotheses were tested. First, RR would increase with increasing T and RH. Second, the temperature-associated increase in RR might differ according to sex and growth stage. Third, given the positive association between body-surface temperature and RR previously reported in heat-stressed and water-deprived goats [25], we hypothesized that flank ST would remain positively associated with RR after adjustment for T, RH, sex, growth stage, and baseline body weight (BW). The RR values of 40, 60, and 80 breaths/min were selected with reference to the respiration rate classification described in [8] and were treated as operational criteria rather than biologically validated heat-stress thresholds.

Therefore, this study had two main objectives: (1) to quantify the associations of T and RH with RR in Korean native black goats and evaluate potential differences according to sex and growth stage using a linear mixed-effects model accounting for repeated measurements and shared environmental and housing conditions; and (2) to assess the additional explanatory contribution of ST and derive preliminary population-level RR prediction equations and model-derived reference temperatures corresponding to the prespecified RR criteria. These objectives were intended to provide preliminary population-level descriptions of RR in a small, repeatedly measured sample rather than to establish universal heat-stress thresholds or definitive subgroup-specific response patterns.

This study provides quantitative field-based information on the associations of T and RH with RR in Korean native black goats while accounting for repeated measurements and shared date- and pen-level conditions. The findings may support the development of practical thermal-monitoring approaches and provide a basis for future validation under broader environmental and management conditions.

## 2. Materials and Methods

### 2.1. Experimental Site, Animals, and Management

The experiment was conducted from 4 August to 10 November 2025 at the Korean native black goat facility of the Gyeongsang National University experimental farm in Jinju, Gyeongsangnam-do, Republic of Korea (35°20′ N, 128°13′ E). The measurement period was extended into the seasonal transition period to obtain observations across a sufficiently broad temperature range for modeling RR responses from relatively mild to high-temperature conditions.

A total of 24 clinically healthy Korean native black goats were selected. The animals were classified into four groups according to sex and growth stage: adult males (*n* = 6), growing males (*n* = 6), adult females (*n* = 6), and growing females (*n* = 6). The animals were not randomly allocated to these groups; group membership reflected each animal’s pre-existing sex and age at the beginning of the study. The number of animals was equal between sexes, with 12 males and 12 females. For the purposes of this study, goats were operationally classified as growing (≤6 months of age) or adult (>6 months of age), with the 6-month cutoff selected with reference to reported reproductive maturation around this age in Korean native goats [26,27]. Growth-stage classification was determined based on each animal’s age at the beginning of the experimental period and remained unchanged throughout the study, even if a growing goat exceeded 6 months of age during the measurement period. The growing goats used in this study were 5-month-old males (16.0 ± 1.3 kg) and 5-month-old females (10.8 ± 1.0 kg), while the adult goats were 24-month-old males (38.9 ± 1.7 kg) and 24-month-old females (24.1 ± 6.9 kg). Individual BW was measured once at the beginning of the experimental period and was used as the baseline BW for each animal throughout the study.

The experiment was conducted in a conventional black goat house with walls and upper wall openings for natural ventilation. Each pen had a total area of 2.3 m × 7.7 m and was divided into a resting area (2.3 m × 3.0 m) and an exercise area (2.3 m × 4.7 m), with a doorway allowing the goats to move freely between the two areas. Both areas were roofed and enclosed by walls, with ventilation openings located only in the upper wall sections above the level normally occupied by the goats. The stated dimensions refer to the total area of each pen rather than the space per individual animal. Adult males were housed individually in six separate pens (pens 1–6). Growing males were distributed across pens 7, 8, and 11 (1, 2, and 3 animals, respectively), adult females across pens 7, 8, and 9 (2 animals per pen), and growing females across pens 7, 8, and 10 (2, 1, and 3 animals, respectively). Thus, no sex-by-growth-stage group was housed entirely within a single pen. Pen was included as a random intercept in the mixed-effects models to account for housing conditions shared by animals within the same pen.

For feeding management, a commercial pelleted concentrate and timothy hay were each provided at 2% of BW on an as-fed basis, twice daily at 09:10 and 14:30. According to the manufacturer’s feed label, the pelleted concentrate contained ≥0.8% calcium and ≤0.8% phosphorus, with a total digestible nutrient (TDN) content of 67%. Individual feed intake was not measured; therefore, the stated amounts represent feed offered rather than actual consumption. Feed was not available immediately before or during physiological measurements; the afternoon feeding was provided only after all ST and RR measurements had been completed. Drinking water was provided continuously ad libitum through water troughs installed outside the barn.

The chemical compositions of timothy hay and commercial pelleted concentrate were analyzed. Moisture content was determined by drying feed samples in a dry oven (OF-22GW, Jeio Tech, Seoul, Republic of Korea) at 55 °C for 48 h, and dry matter (DM) content was calculated by subtracting moisture content from 100%. Crude ash (CA) was determined using a muffle furnace at 550 °C for 5 h. Crude protein (CP) was determined by the Kjeldahl method (AOAC method 984.13) [28] using an N analyzer (B-324, 412, 435, and 719 S Titrino; BUCHI, Flawil, Switzerland). Ether extract (EE) was analyzed using the Soxhlet method. Neutral detergent fiber (NDF) and acid detergent fiber (ADF) were analyzed according to AOAC methods 2002.04 and 973.18, respectively [28], using an ANKOM200 fiber analyzer (ANKOM Technology, Macedon, NY, USA). The DM contents of timothy hay and commercial pelleted concentrate were 90.68% and 88.40%, respectively. On a DM basis, the CP, EE, CA, NDF, and ADF contents of timothy hay were 10.70%, 2.37%, 10.60%, 61.90%, and 39.50%, respectively. The corresponding values for commercial pelleted concentrate were 16.50%, 3.12%, 7.37%, 31.80%, and 15.40%, respectively.

The experimental protocol was approved by the Institutional Animal Care and Use Committee (IACUC) of Gyeongsang National University (GNU-250623-E0129; approved 23 June 2025), and all animal procedures were conducted in accordance with applicable institutional animal care and welfare guidelines.

### 2.2. Measurements and Data Collection

The study was conducted over a temperature range of 15–33 °C. Measurements were performed on 35 dates across seven predefined target temperature conditions at 3 °C intervals, approximately corresponding to 15, 18, 21, 24, 27, 30, and 33 °C. Five measurement dates were selected for each target temperature condition based on the ambient temperature observed at 14:00. The same 24 goats were repeatedly measured on 35 non-consecutive dates between 4 August and 10 November 2025. Accordingly, each temperature category included 30 animal-date observations per sex-by-growth-stage group (five measurement dates × six goats), resulting in 210 repeated observations per group and 840 animal-date observations in total. Each observation was identified by animal identity and measurement date. Measurements were standardized at 14:00, when relatively high daytime temperatures were expected, to obtain comparable observations across the predefined thermal conditions while minimizing variation attributable to measurement time.

Environmental variables included T, RH, and radiant energy (RE). T and RH were measured using a portable digital thermo-hygrometer (Testo 480; Testo SE & Co. KGaA, Titisee-Neustadt, Germany) positioned at a height corresponding to the goats’ head level. RE was measured using a portable solar meter (TM-751; Tenmars Electronics Co., Ltd., Taipei, Taiwan). T and RH were measured once on each measurement date at goat head level in the central area of the barn and were not recorded continuously. The resulting date-specific T and RH values were linked to the physiological observations obtained from all animals on the corresponding measurement date. Environmental measurements were obtained in shaded locations beneath the barn roof to reflect the thermal conditions experienced by the goats within the housing environment. The barn roof was insulated, and measured RE remained low, ranging from 1 to 7 W/m^2^, with limited variation across measurement dates. Consequently, RE was not retained in the final statistical model. Wind speed was not systematically recorded on each measurement date. However, measurements conducted in rooms with the same structural configuration indicated low air movement, generally ranging from 0.1 to 0.3 m/s. Given the low air movement and the location of the ventilation openings above the level normally occupied by the goats, wind speed was not included as an environmental predictor in the statistical model.

Physiological variables included ST and RR. ST was measured at the right flank using an infrared thermal imaging camera (Testo 868; Testo SE & Co. KGaA, Titisee-Neustadt, Germany). Thermal images were acquired when the animal was stationary, from a distance of approximately 1.0–1.5 m, with the camera lens positioned as close as possible to perpendicular to the flank surface. The emissivity was maintained at 0.95. Areas with wet hair or visible foreign material on the hair coat were avoided. Measurements were conducted beneath the barn roof without direct solar exposure. The right flank was selected as a standardized body-surface site because flank temperature has been used in previous thermographic studies of ruminants and has been reported to be strongly associated with the temperature–humidity index (THI) [29]. Using a single standardized anatomical site also reduced variation attributable to measurement location and allowed repeated measurements to be performed consistently across animals and dates. This site was also selected to improve measurement repeatability and field applicability. RR was determined by visually counting vertical movements of the right flank for 1 min while the animal remained as calm as possible and was expressed as breaths per minute. Each animal’s RR was measured once on each measurement date. On each measurement date, RR measurements were conducted by three trained observers using the same standardized 1 min counting protocol. The observers were not blinded to animal group or environmental conditions because of the field-based study design. To minimize disturbance associated with human approach or animal movement, ST and RR were measured without relocating or restraining the goats, at the location each animal occupied at the time of observation. No standardized resting period or posture was imposed before measurement; the goats were assessed in the naturally occurring posture and activity state at the time of observation. All animals were assessed at the standardized measurement time of 14:00 using the same measurement protocol.

### 2.3. Statistical Analysis

Descriptive statistical analyses were conducted using SPSS Statistics software (version 27.0; IBM Corp., Armonk, NY, USA), and mixed-effects analyses were performed using R software (version 4.6.1; R Foundation for Statistical Computing, Vienna, Austria). All tests were two-sided, and statistical significance was declared at *p* < 0.05. Descriptive data are presented as the mean ± standard deviation (SD), minimum, and maximum.

Because RR exhibited a right-skewed distribution and increasing variability at higher values, RR was transformed using the natural logarithm before mixed-effects analysis. T was centered at 24 °C, corresponding to the mean temperature during the study period. RH, BW, and ST were centered at their respective overall means. Sex and growth stage were treated as categorical fixed factors using sum-to-zero contrasts. Female and male goats were coded as 1 and −1, respectively, while adult and growing goats were coded as 1 and −1, respectively.

Repeated measurements of RR were analyzed using a linear mixed-effects model. Although the dataset comprised 840 repeated animal-date observations, T and RH were measured at the date level; therefore, inference regarding their associations with RR was based primarily on between-date variation across the 35 measurement dates rather than on 840 independent environmental observations. The initial full fixed-effects structure included sex, growth stage, centered temperature, and all corresponding two- and three-way interactions. Centered RH and BW were included as additional fixed effects. To account for repeated measurements and individual heterogeneity in temperature responses, the model included an animal-specific random intercept and random slope for centered temperature. The intercept-slope covariance was constrained to zero because the unconstrained model estimated a near-boundary correlation and a singular fit when fitted using maximum likelihood, while allowing the covariance did not materially alter the fixed-effect estimates or substantive conclusions. The random-slope structure was evaluated both before and after fixed-effects selection using information criteria and model diagnostics. Measurement date and pen identity were additionally included as random intercepts to account for conditions shared by all animals measured on the same date and housed in the same pen, respectively.

Sex, growth stage, temperature, RH, and body weight were retained as fixed effects in all candidate models. Model selection was applied only to the two- and three-way interactions among sex, growth stage, and temperature. The same random-effects structure was maintained across all candidate fixed-effects models. Hierarchical candidate models were fitted using maximum likelihood and compared using likelihood-ratio tests, Akaike’s information criterion (AIC), Bayesian information criterion (BIC), and model parsimony. The selected model was subsequently refitted using restricted maximum likelihood for coefficient estimation. Type III fixed-effect tests were performed using Satterthwaite’s approximation for denominator degrees of freedom [30]. All linear mixed-effects models were fitted using the lmer() function in the lmerTest package, which extends the lme4 framework, and Type III fixed-effect tests were obtained using the anova() function in lmerTest.

ST was evaluated as a secondary analysis because, unlike the date-level environmental variables T and RH, it required separate animal-level measurement using infrared thermography. The purpose of this analysis was to determine whether ST provided additional explanatory information about RR beyond the variables included in the primary environmental model. The association between ST and RR was evaluated by adding centered ST to the primary model while retaining the same covariates and random-effects structure. The primary and ST-extended models were fitted using maximum likelihood and compared using likelihood-ratio tests, AIC, and BIC. Candidate interactions of ST with sex and growth stage were also evaluated using the same model-comparison criteria. RE was examined descriptively but was not included in the final mixed-effects models because of its restricted variation under the shaded housing conditions.

Model assumptions were assessed using quantile–quantile plots of the conditional residuals and plots of conditional residuals against fitted values. Model convergence and singularity were also examined. Potential residual temporal dependence was assessed using an empirical semivariogram of within-animal conditional residuals based on the actual number of days separating measurement dates [31]. Fixed-effect estimates from the log-transformed models were exponentiated and reported as response ratios with 95% confidence intervals. Marginal and conditional coefficients of determination were calculated to describe the variance explained by the fixed effects alone and by the combined fixed and random effects, respectively [32].

Using the prespecified RR criteria of 40, 60, and 80 breaths/min, model-derived reference temperatures were estimated from the present data. Their correspondence to distinct biological levels of heat-stress severity in Korean native black goats was not independently validated using additional physiological, behavioral, welfare, or production indicators. Reference temperatures were obtained for each sex-by-growth-stage group from the population-level fixed-effect predictions of the final primary mixed-effects model, with RH and BW fixed at their overall means. Predicted values were back-transformed from the logarithmic scale and represent fixed-effect predictions on the median (geometric mean) scale, with animal-, date-, and pen-specific random effects set to zero. The 95% confidence intervals for the reference temperatures were obtained using 1000 parametric bootstrap replicates. Parametric bootstrapping was performed using the bootMer() function in the lme4 package. Reference-temperature estimates outside the observed temperature range of 15–33 °C were considered extrapolated values and were not interpreted as directly observed experimental values.

## 3. Results

### 3.1. Environmental and Physiological Conditions During the Experimental Period

Descriptive statistics for the environmental and physiological variables measured during the experimental period are presented in Table 1. Across the 35 measurement dates, temperature ranged from 15 to 33 °C, with a mean of 24.00 ± 6.09 °C. Relative humidity ranged from 25.0 to 70.5%, and RE under the shaded barn conditions ranged from 1 to 7 W/m^2^. Across the 840 animal-date observations, skin temperature ranged from 20.5 to 39.0 °C, with a mean of 30.64 ± 4.82 °C. RR showed considerable variation, ranging from 11 to 150 breaths/min, with a mean of 56.58 ± 34.30 breaths/min.

**Figure 1:**
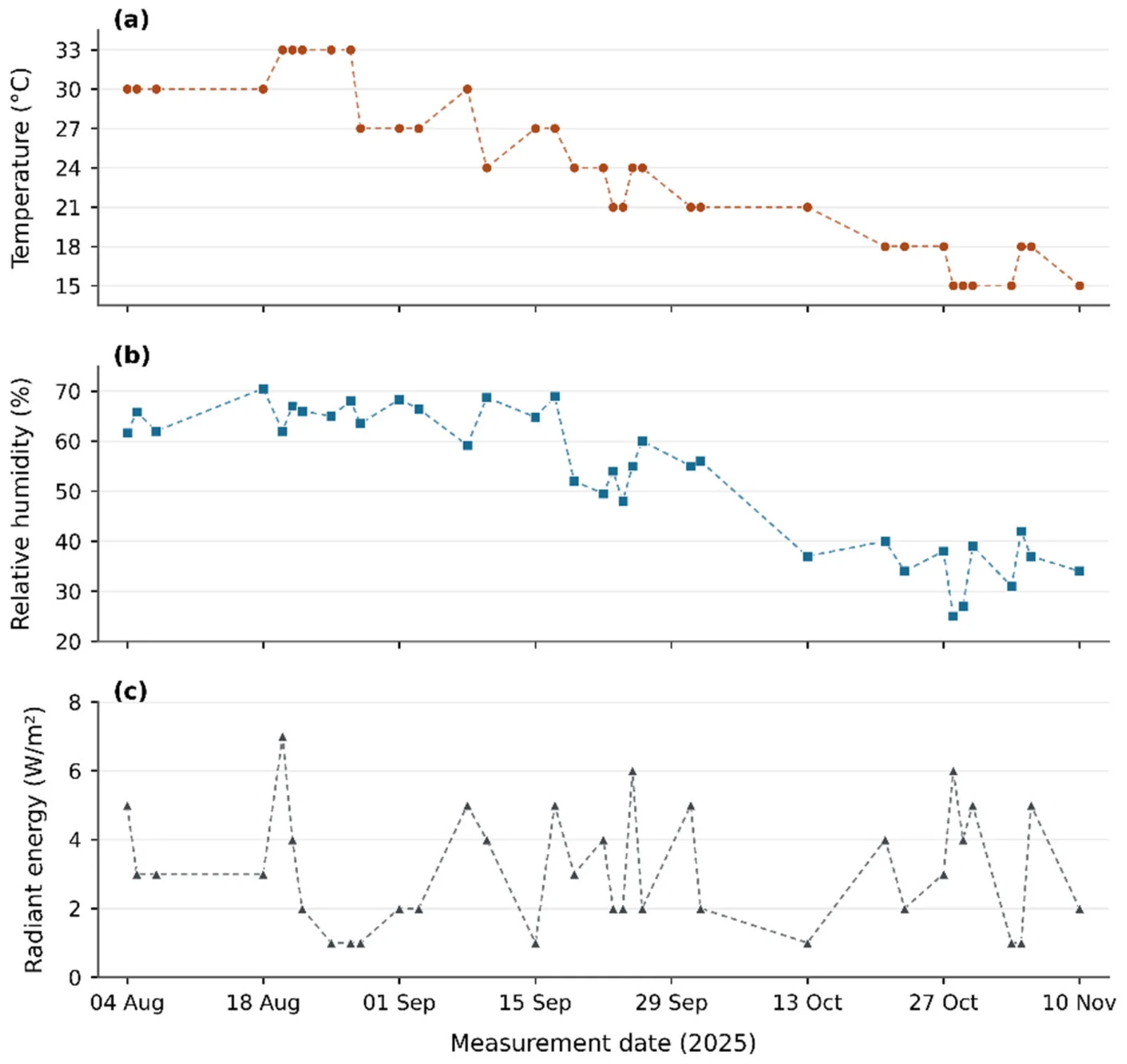
Temporal variation in environmental conditions across the 35 measurement dates. (**a**) Ambient temperature (°C); (**b**) relative humidity (%); and (**c**) radiant energy (W/m^2^). Environmental variables were recorded at 14:00 on each measurement date. Dashed lines connect the observed values for visualization and do not represent continuous monitoring.

The temporal variation in ambient temperature, relative humidity, and radiant energy across the 35 measurement dates is illustrated in Figure 1.

### 3.2. Effects of Temperature, Relative Humidity, Sex, and Growth Stage on Respiration Rate

The random-effect variance components of the selected primary linear mixed-effects model are presented in Table 2. Starting from the full model containing all two- and three-way interactions among sex, growth stage, and temperature, candidate interaction structures were compared while retaining the main effects of sex, growth stage, T, RH, and BW. The selected model had a slightly lower AIC than the full interaction model (−562.97 vs. −562.40) and a considerably lower BIC (−506.17 vs. −491.40). A likelihood-ratio test showed that adding the sex × T, growth stage × T, and sex × growth stage × T interactions did not significantly improve model fit ($\chi_{3}^{2}=5.43$, *p* = 0.143). The full and selected models had the same marginal and conditional *R*^2^ values ($R_{m}^{2}=0.916$ and $R_{c}^{2}=0.940$, respectively), indicating that the additional interaction terms did not materially increase the explained variance. Thus, the additional temperature-related interactions did not provide sufficient improvement in model fit to justify their additional complexity.

The animal-specific random slope for temperature was retained in the selected model. Compared with the random-intercept-only model, the random-slope model had a lower AIC (−562.97 vs. −559.98), although its BIC was slightly higher (−506.17 vs. −507.91). The likelihood-ratio test indicated improved model fit after inclusion of the random slope ($\chi_{1}^{2}=5.00$, *p* = 0.025). The marginal and conditional *R*^2^ values were 0.916 and 0.940 for the random-slope model, compared with 0.915 and 0.939 for the random-intercept-only model. Based on the AIC, likelihood-ratio test, and the prespecified consideration of individual heterogeneity in temperature responses, the random-slope structure was retained in the final model. The estimated random-effect variance components of the selected model are presented in Table 2.

**Figure 2:**
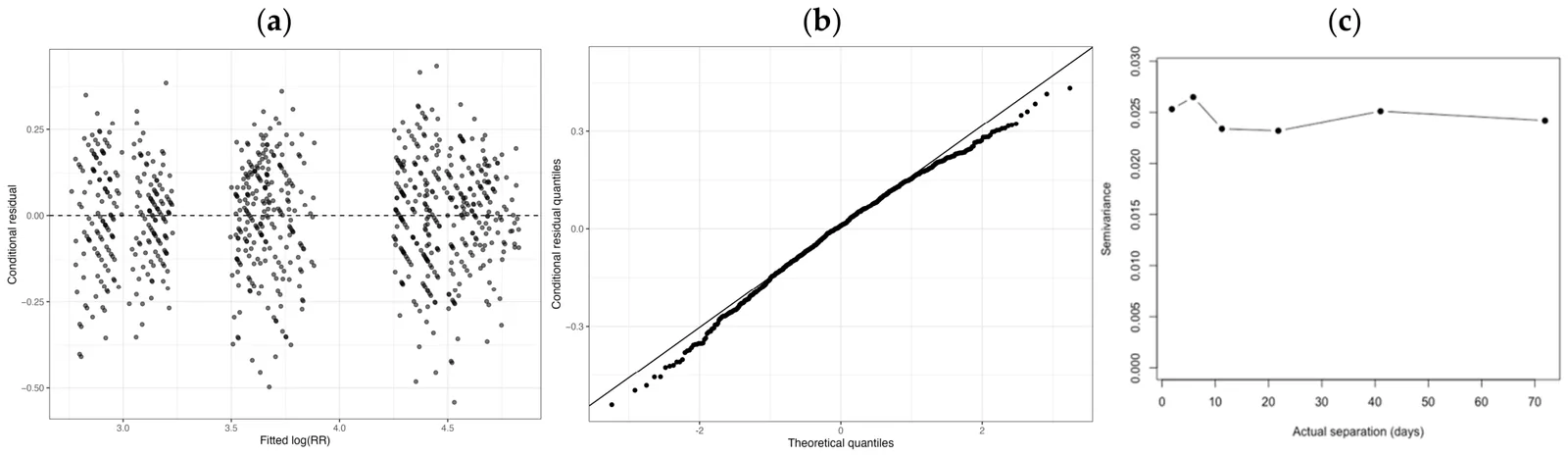
Diagnostic plots for the selected primary linear mixed-effects model. (**a**) Conditional residuals plotted against fitted values on the log-RR scale. (**b**) Normal Q-Q plot of the conditional residuals. (**c**) Empirical semivariogram of within-animal conditional residuals according to the actual number of days separating measurement dates.

Diagnostic plots for the selected primary model are presented in Figure 2. The conditional residuals were centered around zero and showed no pronounced systematic trend or substantial change in dispersion across the fitted values (Figure 2a). The Q-Q plot followed the reference line closely over most of the distribution, although modest deviations were observed in the tails (Figure 2b). Because the measurement dates were irregularly spaced, temporal dependence was evaluated using the actual number of days separating observations. The empirical semivariogram of the within-animal conditional residuals remained approximately constant across temporal separations, providing no clear evidence of residual temporal autocorrelation (Figure 2c). Overall, the diagnostic plots did not indicate major systematic violations of the model assumptions.

Temperature was positively associated with RR (*F*_1,33.06_ = 186.18, *p* < 0.001). A 1 °C increase in temperature was associated with an estimated 9.23% increase in RR (response ratio = 1.092; 95% CI: 1.078–1.107). RH was also positively associated with RR (*F*_1,32.00_ = 7.93, *p* = 0.008). Each 1 percentage-point increase in RH was associated with an estimated 0.79% increase in RR (response ratio = 1.008; 95% CI: 1.002–1.014). These estimates describe associations within the environmental ranges observed in this study (15–33 °C and 25.0–70.5% RH). BW was not significantly associated with RR (*p* = 0.422) but served as an adjustment covariate to account for individual differences in body size.

After adjustment for T, RH, and BW, growth stage was significantly associated with RR (*F*_1,13.13_ = 7.69, *p* = 0.016). Averaged across sex, adult goats had an estimated 13.05% higher RR than growing goats (response ratio = 1.130; 95% CI: 1.028–1.244). The overall effect of sex was not statistically significant (*F*_1,17.95_ = 3.10, *p* = 0.095). Although the sex × growth stage interaction was retained in the selected model based on the model-selection criteria, it did not reach the conventional level of statistical significance (*F*_1,16.39_ = 3.76, *p* = 0.070). Accordingly, evidence that the association between sex and RR differed according to growth stage was limited. The Type III fixed-effect test results for the selected model are summarized in Table 3.

Respiration rate was analyzed after natural-log transformation. Temperature was centered at 24 °C, and relative humidity and body weight were centered at their overall means. The model included fixed effects of sex, growth stage, temperature, interaction between sex and growth stage, and relative humidity and body weight as covariates. An animal-specific random intercept, temperature slope, and random intercepts for measurement date and pen identity were included.

### 3.3. Association Between Skin Temperature and Respiration Rate

The results of the linear mixed-effects model including ST are presented in Table 4. This model retained the fixed- and random-effects structure of the selected primary model, with ST added as an additional fixed effect. ST remained positively associated with RR after adjustment for T, RH, BW, sex, growth stage, and the interaction between sex and growth stage (*F*_1,816.24_ = 34.28, *p* < 0.001). A 1 °C increase in ST was associated with an estimated 3.08% increase in RR (response ratio = 1.031; 95% CI: 1.020–1.041).

Adding ST to the primary model reduced the AIC from −562.97 to −594.89 and the BIC from −506.17 to −533.35. The likelihood-ratio test also showed that adding ST significantly improved model fit ($\chi_{1}^{2}=33.91$, *p* < 0.001). The marginal *R*^2^ increased modestly from 0.916 to 0.923, while the conditional *R*^2^ increased from 0.940 to 0.942. These results indicate that ST provided additional in-sample explanatory information about RR beyond the variables included in the primary model. However, this improvement does not establish externally validated predictive performance or diagnostic utility for thermal strain.

The full ST interaction model had a slightly lower AIC than the additive ST model (−595.82 vs. −594.89) but a substantially higher BIC (−520.09 vs. −533.35). In addition, the ST-related interactions did not significantly improve model fit ($\chi_{3}^{2}=6.94$, *p* = 0.074). Therefore, the more parsimonious additive ST model was selected, and there was no clear evidence that the adjusted association between ST and RR differed according to sex or growth stage.

### 3.4. Population-Level Prediction Equations for Respiration Rate

Population-level RR prediction equations were derived from the fixed-effect estimates of the selected primary linear mixed-effects model. The integrated fixed-effect prediction equation was expressed as follows:

$$\hat{\mathrm{RR}}=\exp\left[3.8335-0.0236\mathrm{Sex}+0.0613\mathrm{Growth}\;\mathrm{Stage}+0.0883T_{c}+0.0079RH_{c}-0.0019BW_{c}-0.0180\left(\mathrm{Sex}\times \mathrm{Growth}\;\mathrm{Stage}\right)\right],$$

where *T_c_* = *T* − 24, *RH_c_* = *RH* − 53.18, and *BW_c_* = *BW* − 22.45. Note that although the sex × growth stage interaction did not reach the conventional level of statistical significance, it was retained because the fixed-effects structure was selected at the model level using AIC and BIC.

Substitution of the corresponding sex and growth-stage codes into the integrated equation produced the group-specific prediction equations presented in Table 5. The group-specific equations were derived from a single integrated mixed-effects model rather than from separate regression analyses for each group. Animal-, date-, and pen-specific random effects were set to zero when calculating population-level predictions. Consequently, the back-transformed estimates represent population-level conditional median RR values on the original scale. The analysis did not provide clear evidence that temperature-associated RR slopes differed by sex or growth stage. Given the limited number of animals per group (*n* = 6), the group-specific predictions should be interpreted as descriptive outputs of the fitted model rather than confirmed subgroup-specific thermal response profiles.

### 3.5. Model-Derived Reference Temperatures Corresponding to Respiration Rate Criteria

**Figure 3:**
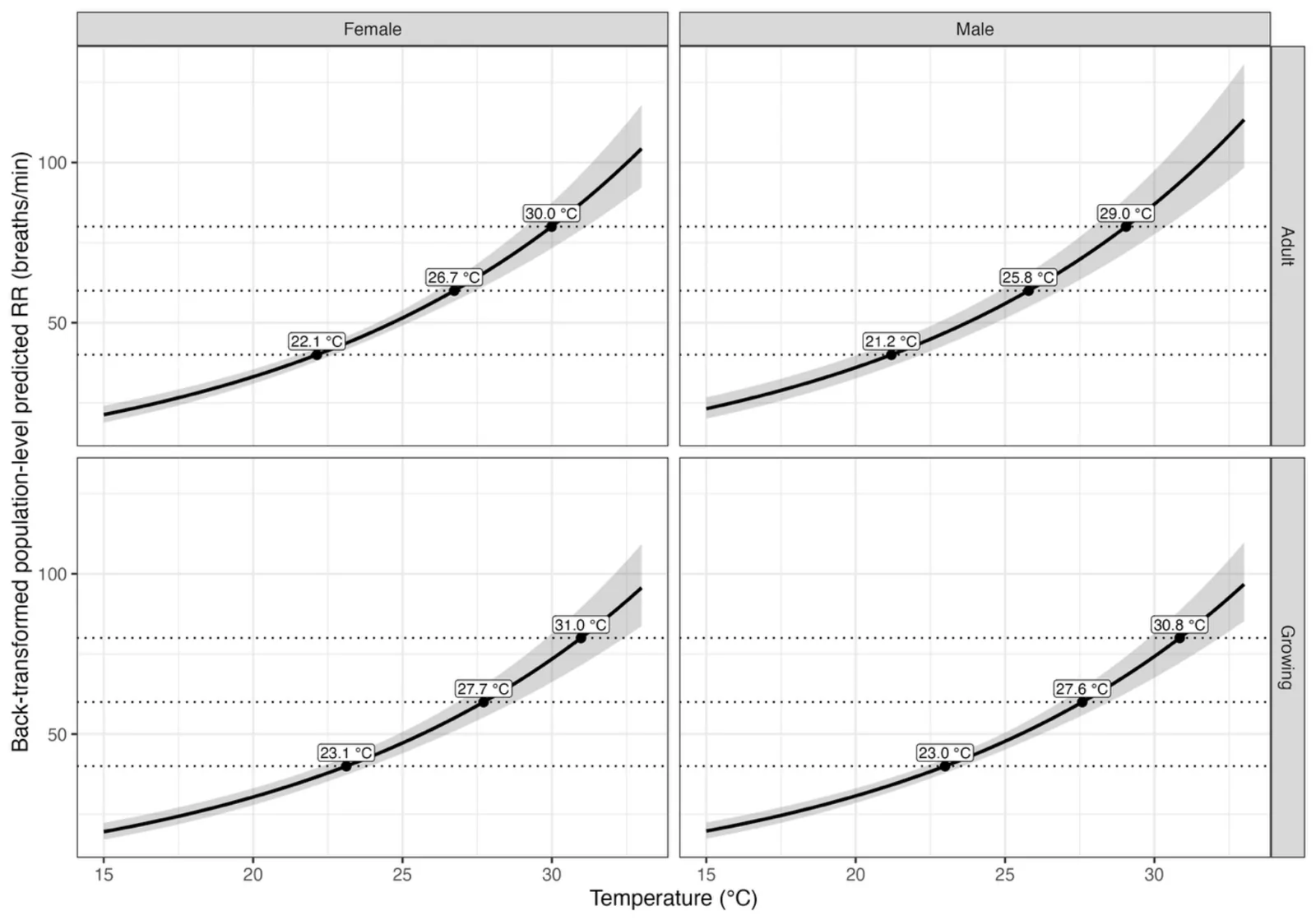
Population-level predicted respiration rate and model-derived reference temperatures according to temperature in adult and growing female and male Korean native black goats. Horizontal dotted lines indicate RR criteria of 40, 60, and 80 breaths/min. Points and accompanying labels indicate the estimated temperatures at which the predicted RR reached each criterion. Predictions were derived from the fixed effects of the selected primary linear mixed-effects model, with RH and BW fixed at their overall means. Shaded areas represent 95% confidence intervals.

Based on the integrated population-level prediction equations, the temperatures corresponding to predicted RR of 40, 60, and 80 breaths/min were estimated for each sex-by-growth-stage group (Table 6 and Figure 3). These model-derived reference temperatures were calculated from the fixed-effect predictions of the selected primary linear mixed-effects model, with RH and BW fixed at their overall means.

For adult females, predicted RR values of 40, 60, and 80 breaths/min were reached at 22.14 °C, 26.73 °C, and 29.99 °C, respectively. The corresponding reference temperatures for adult males were 21.20 °C, 25.79 °C, and 29.05 °C. In growing females, the estimated reference temperatures were 23.12 °C, 27.71 °C, and 30.97 °C, whereas those for growing males were 22.99 °C, 27.59 °C, and 30.85 °C.

Adult males showed numerically lower model-derived reference temperatures than the other groups across all three RR criteria. However, these estimates were obtained for each group from the same integrated mixed-effects model, and formal between-group differences in reference temperature were not established. All point estimates and their 95% confidence intervals were within the observed temperature range of 15–33 °C.

Model-derived reference temperatures were estimated from back-transformed population-level fixed-effect predictions of the final primary linear mixed-effects model, with relative humidity and body weight fixed at their overall means. The predicted values represent fixed-effect predictions on the median (geometric mean) scale, with animal-, date-, and pen-specific random effects set to zero.

## 4. Discussion

This single-farm study of 24 Korean native black goats quantified RR responses under field conditions over the observed T range of 15–33 °C and RH range of 25.0–70.5% while accounting for repeated measurements within animals and shared date- and pen-level conditions. T and RH were positively associated with RR, and adult goats had a higher adjusted RR than growing goats. However, the available data did not provide clear evidence that the slopes of the association between temperature and RR differed according to sex or growth stage. ST was also positively associated with RR after adjustment for environmental and animal-related factors and provided modest additional in-sample explanatory information. The fitted model was subsequently used to derive population-level prediction equations and model-derived reference temperatures corresponding to RR criteria of 40, 60, and 80 breaths/min. These findings describe statistical associations and preliminary population-level reference points within the conditions represented in this study. Both the model-derived reference temperatures and the additional explanatory value of ST are context-dependent; the reference temperatures should not be interpreted as validated biological boundaries for heat-stress severity or welfare impairment.

### 4.1. Associations of Temperature and Relative Humidity with Respiration Rate

Within the observed T range of 15–33 °C, each 1 °C increase in T was associated with an estimated 9.23% increase in RR (response ratio = 1.092; 95% CI: 1.078–1.107). This direction of response is physiologically consistent with the increased reliance of ruminants on respiratory evaporative heat loss as environmental heat load increases and sensible heat dissipation becomes less effective [4,8]. Controlled studies in dairy goats have similarly reported increases in RR and other thermoregulatory responses during heat exposure [17,18], while increased RR under thermal stress has also been reported in native and crossbred goats [33]. More recent studies in other goat breeds have likewise reported increases in RR together with changes in rectal or surface temperature and other physiological, ingestive, or metabolic responses under elevated thermal conditions [14,15,34]. Direct numerical comparisons among studies are limited because breed, production stage, exposure protocol, climatic conditions, and measures of heat load differ; nevertheless, the consistent direction of response supports RR as a sensitive field indicator of increasing thermal load in goats.

RH was also positively associated with RR. Within the observed RH range of 25.0–70.5%, each 1 percentage-point increase in RH was associated with an estimated 0.79% increase in RR (response ratio = 1.008; 95% CI: 1.002–1.014) after adjustment for T and the other model terms. Increased atmospheric moisture can reduce the vapor-pressure gradient supporting evaporative heat loss and thereby reduce the efficiency of respiratory cooling [35,36]. The positive association of RH with RR therefore indicates that RH should be considered together with T when interpreting respiratory responses under field conditions. However, T and RH were measured at the measurement-date level, and the corresponding coefficients reflect associations across the 35 measurement dates rather than causal effects estimated from 840 independent environmental observations.

The estimated associations of T and RH should therefore be interpreted within the environmental ranges represented in this study. They do not demonstrate that a particular T, RH, or RR value corresponds to a specific degree of physiological strain, reduced productivity, or welfare impairment. Such biological interpretation would require concurrent assessment of independent outcomes such as core body temperature, behavior, feed and water intake, physiological or metabolic variables, productivity, and welfare. The present results describe environmental associations with RR rather than biologically validated levels of heat-stress severity.

### 4.2. Sex, Growth Stage, and Individual Variation in Respiration Rate

After adjustment for T, RH, and baseline BW, adult goats had an estimated 13.05% higher RR than growing goats when averaged across sex (response ratio = 1.130; 95% CI: 1.028–1.244). This finding concerns the adjusted RR level rather than the temperature-response slope. Differences in body size, body composition, metabolism, or thermoregulatory capacity could contribute to variation between growth stages [9,16], but these mechanisms were not directly measured in the present study. The observed association should therefore be interpreted as a difference in modeled RR level rather than evidence of a specific underlying physiological mechanism. BW was included as an adjustment covariate, and its nonsignificant association with RR should likewise not be interpreted as evidence that body size has no biological relevance to thermoregulation.

The overall effect of sex was not statistically significant, and the sex × growth stage interaction provided only limited evidence of group-specific differences. Furthermore, adding the sex × T, growth stage × T, and sex × growth stage × T interactions did not provide sufficient improvement in model fit to justify their inclusion in the selected model. These findings do not demonstrate equivalence among the four sex-by-growth-stage groups. With only six independent animals in each group, the precision and statistical power to detect modest group-specific differences were limited. The appropriate interpretation is therefore that the present data did not provide clear evidence of different temperature-associated RR responses according to sex or growth stage, while potentially meaningful differences cannot be excluded.

Individual variation was also substantial. Observed RR ranged from 11 to 150 breaths/min, and an animal-specific random temperature slope was retained in the selected model, indicating heterogeneity in temperature responses among individual goats. Thus, even though a common population-level temperature association adequately described the data after model selection, individual goats did not necessarily respond identically. These findings emphasize that population-level estimates should not be applied as rigid cut-offs to individual animals.

### 4.3. Additional Information Provided by Skin Temperature

ST was positively associated with RR after adjustment for T, RH, BW, sex, growth stage, and the sex × growth stage interaction. Each 1 °C increase in ST was associated with an estimated 3.08% increase in RR (response ratio = 1.031; 95% CI: 1.020–1.041). Adding ST reduced the AIC from −562.97 to −594.89 and increased marginal R^2^ from 0.916 to 0.923, with the likelihood-ratio test also indicating improved model fit. Thus, ST provided additional explanatory information about RR beyond the variables included in the primary environmental model. However, the increase in explained variance was modest, and this comparison demonstrates additional in-sample explanatory value rather than externally validated predictive performance.

The observed association is physiologically plausible because surface temperature reflects peripheral heat exchange and may change concurrently with increased respiratory heat dissipation [22]. Recent work in heat-stressed goats has also shown that infrared thermographic surface temperature is associated with multiple body-thermal variables, supporting its potential as a complementary noninvasive measure of thermal status [25]. Infrared thermography also permits repeated, non-contact measurement under farm conditions. Nevertheless, ST was measured only at the right flank and represents local surface temperature rather than core body temperature. ST may be influenced by peripheral blood flow, hair-coat characteristics, posture, air movement, and measurement conditions, and both ST and RR may increase in response to the same underlying thermal exposure. Consequently, the observed association should not be interpreted as evidence that increased ST directly causes increased RR.

The ST-related interactions with sex and growth stage did not provide sufficient improvement in model fit to be retained. As with the temperature-related interactions, this finding should not be interpreted as demonstrating identical ST–RR relationships across groups, particularly given the limited number of animals per group. ST is therefore more appropriately regarded as a complementary animal-level indicator than as a stand-alone diagnostic measure. RR and ST together also do not constitute a complete assessment of heat stress and should be interpreted in conjunction with environmental conditions, animal behavior, core-temperature information when available, and the condition of the individual animal.

### 4.4. Population-Level Prediction Equations and Model-Derived Reference Temperatures

The group-specific RR prediction equations were derived from a single integrated mixed-effects model rather than from separate regression analyses for each group. This approach allowed the common associations of T and RH and the tested group terms to be estimated while accounting for repeated measurements within animals and conditions shared at the measurement-date and pen levels. Because the model was fitted to natural-log-transformed RR, the back-transformed equations describe multiplicative changes on the original scale. The equations represent population-level fixed-effect predictions of median RR, with animal-, date-, and pen-specific random effects set to zero, and should therefore not be interpreted as individualized prediction equations.

With RH and BW fixed at their overall means, the model-derived reference temperatures corresponding to RR criteria of 40, 60, and 80 breaths/min ranged across groups from 21.20 to 23.12 °C, 25.79 to 27.71 °C, and 29.05 to 30.97 °C, respectively. All point estimates and their 95% confidence intervals were within the observed T range of 15–33 °C. Adult males showed numerically lower reference temperatures than the other groups across the three RR criteria; however, formal between-group differences in the derived reference temperatures were not established. This numerical ordering should therefore remain descriptive and should not be interpreted as evidence that adult males are more susceptible to heat.

The RR values of 40, 60, and 80 breaths/min were used as prespecified operational criteria with reference to the respiration rate classification described in [8]. They were not independently validated in the present study against core body temperature, physiological disruption, production loss, behavior, or welfare outcomes. Studies relating physiological and behavioral responses to thermal indices in goats have proposed different critical ranges, further indicating that heat-stress criteria depend on the environmental metric, breed, and biological outcomes used for their derivation [20,37]. Accordingly, the temperatures estimated in the present study should be interpreted as model-derived reference points for thermal monitoring rather than as universal critical temperatures or validated boundaries separating discrete levels of heat stress.

The reference temperatures are also conditional on the values used for prediction. RH was fixed at its overall mean of 53.18%; therefore, the T corresponding to a given RR criterion may differ under drier or more humid conditions. Furthermore, the wide observed RR range and animal-specific variation in temperature response indicate that an individual goat may reach a given RR at a lower or higher T than the population-level estimate. The equations and reference temperatures may therefore support preliminary screening and identify conditions warranting closer observation, but they should not replace direct assessment of individual animals.

### 4.5. Practical Implications for Thermal Monitoring

Under housing and environmental conditions similar to those studied, repeated RR measurements may support preliminary observation of changing thermal conditions in Korean native black goats. ST may provide complementary animal-level information when infrared thermography is available. However, neither RR nor ST should be used in isolation to determine the severity of heat stress.

The model-derived reference temperatures may help identify circumstances warranting closer animal-level assessment, but should not be used as fixed intervention thresholds or stand-alone indicators of heat-stress severity. Their interpretation should consider concurrent RH, air movement and housing conditions, other sources of environmental heat load, and the response of the individual animal. Repeated RR measurements, supplemented where appropriate by ST and other animal-level observations, may therefore provide complementary information for preliminary thermal monitoring under conditions similar to those represented in the present study.

### 4.6. Limitations and Future Research

Several limitations restrict the generalization and biological interpretation of the present findings. First, the study involved 24 goats at a single experimental farm, with six independent animals in each sex-by-growth-stage group. Although repeated measurements yielded 840 animal-date observations, these observations do not represent 840 independent animals or environmental replicates. T and RH were measured at the date level, and inference regarding their associations with RR was based primarily on between-date variation across 35 measurement dates. The animal-, date-, and pen-level random effects accounted for important sources of dependence, but these modeling procedures do not create additional independent replication or eliminate all limitations associated with the unbalanced pen allocation. The limited number of animals per group also reduced the ability to detect modest sex- or growth-stage-specific differences.

Second, measurements were standardized at 14:00 to obtain comparable observations across the predefined thermal conditions. This approach minimized variation attributable to measurement time but did not characterize complete diurnal patterns of heat accumulation and recovery. T and RH were measured once per measurement date in a central barn location rather than continuously, and air movement was not systematically recorded on each study date. RE remained low under the insulated barn roof, ranging from 1 to 7 W/m^2^. Activity state and posture were not standardized before physiological measurements, although animals were observed without relocation or restraint to minimize handling-related disturbance. These field conditions may have contributed to residual environmental and animal-level variation.

Third, ST was measured only at the right flank. Although the use of a standardized anatomical site improved measurement consistency, ST can vary among body regions and can be affected by hair characteristics, peripheral blood flow, posture, and local environmental conditions. Measurements from multiple anatomical sites together with core body temperature would provide a more comprehensive characterization of animal thermal status.

Fourth, the study did not simultaneously assess core body temperature, behavior, feed and water intake, blood variables, productive performance, or validated welfare outcomes. This limitation is particularly important for interpretation of the RR criteria of 40, 60, and 80 breaths/min. The present analysis identifies statistical associations and model-derived population-level reference values but cannot determine whether a particular RR corresponds to a specific degree of physiological or welfare impairment.

Finally, the prediction equations and model-derived reference temperatures require external validation before broader application. Future studies should include larger populations across multiple farms, housing systems, seasons, and climatic regions; repeated or continuous environmental measurements; observations across different periods of the day; and independent physiological, behavioral, production, and welfare outcomes. Such studies would permit more precise evaluation of group-specific responses and determine whether combinations of RR, ST, environmental variables, and additional animal-level measures improve the early identification of meaningful thermal strain.

## 5. Conclusions

Within the observed temperature range of 15–33 °C and RH range of 25.0–70.5%, T and RH were positively associated with RR in 24 Korean native black goats repeatedly measured at a single experimental farm. Adult goats had a higher adjusted RR than growing goats, whereas the present data did not provide clear evidence that the slopes of the association between temperature and RR differed according to sex or growth stage. Flank ST was also positively associated with RR after adjustment for environmental and animal-related factors and provided modest additional in-sample explanatory information. Model-derived temperatures corresponding to RR criteria of 40, 60, and 80 breaths/min ranged from 21.20 to 23.12, 25.79 to 27.71, and 29.05 to 30.97 °C, respectively, with RH and baseline BW fixed at their overall means. These values should be regarded as preliminary population-level reference points rather than biologically validated thresholds of heat-stress severity or welfare impairment. RR, together with ST and environmental measurements, may therefore provide complementary information for preliminary thermal monitoring under similar housing and environmental conditions; however, external validation in larger populations across multiple farms and environmental conditions, using independent physiological, behavioral, welfare, and production outcomes, is required before broader application of these model-derived reference points.

## Figures and Tables

**Table 1:** Descriptive statistics for environmental and physiological variables measured during the experimental period.

Variable	Observation Level	Minimum	Mean ± SD	Maximum
Temperature (°C)	35 dates	15.0	24.00 ± 6.09	33.0
Relative humidity (%)	35 dates	25.0	53.18 ± 13.97	70.5
Radiant energy (W/m^2^)	35 dates	1.0	3.11 ± 1.71	7.0
Skin temperature (°C)	840 animal-date observations	20.5	30.64 ± 4.82	39.0
Respiration rate (breaths/min)	840 animal-date observations	11	56.58 ± 34.30	150

Environmental variables were recorded at the measurement-date level and linked to each animal observed on the corresponding date. Skin temperature and respiration rate were measured repeatedly in 24 animals over 35 measurement dates, yielding 840 animal-date observations.

**Table 2:** Random-effect variance components from the selected primary linear mixed-effects model.

Grouping Factor	Random Term	Variance	SD
Animal	Intercept	0.00047	0.02178
Animal	Temperature slope	0.00002	0.00415
Measurement date	Intercept	0.01004	0.10021
Pen	Intercept	0.00009	0.00956
Residual	-	0.02581	0.16064

**Table 3:** Type III fixed-effect tests from the linear mixed-effects model for respiration rate.

Effect	Numerator df	Denominator df	F-Value	*p*-Value
Sex	1	17.95	3.10	0.095
Growth stage	1	13.13	7.69	0.016
Temperature	1	33.06	186.18	<0.001
Relative humidity	1	32.00	7.93	0.008
Body weight	1	16.354	0.68	0.422
Sex × Growth stage	1	16.391	3.76	0.070

**Table 4:** Association between skin temperature and respiration rate in the linear mixed-effects model including skin temperature.

Variable	Estimate	SE	Response Ratio	95% CI	F-Value	*p*-Value
Skin temperature	0.0304	0.0052	1.031	1.020–1.041	34.28	<0.001

Note: RR was analyzed after natural-log transformation. The response ratio represents the multiplicative change in RR associated with a 1 °C increase in ST. CI, confidence interval; SE, standard error.

**Table 5:** Population-level respiration rate prediction equations derived from the final primary linear mixed-effects model.

Sex	Growth Stage	Population-Level Prediction Equation
Female	Adult	$\hat{\mathrm{RR}}=\exp\left[3.853+0.088\left(T-24\right)+0.008\left(\mathrm{RH}-53.18\right)-0.002(\mathrm{BW}-22.45)\right]$
Male	Adult	$\hat{\mathrm{RR}}=\exp\left[3.936+0.088\left(T-24\right)+0.008\left(\mathrm{RH}-53.18\right)-0.002(\mathrm{BW}-22.45)\right]$
Female	Growing	$\hat{\mathrm{RR}}=\exp\left[3.767+0.088\left(T-24\right)+0.008\left(\mathrm{RH}-53.18\right)-0.002(\mathrm{BW}-22.45)\right]$
Male	Growing	$\hat{\mathrm{RR}}=\exp\left[3.778+0.088\left(T-24\right)+0.008\left(\mathrm{RH}-53.18\right)-0.002(\mathrm{BW}-22.45)\right]$

RR, respiration rate in breaths/min; T, temperature in °C; RH, relative humidity in %. The equations represent population-level fixed-effect predictions and exclude animal-, date-, and pen-specific random effects. Predictions should be interpreted within the environmental range observed in the present study.

**Table 6:** Estimated temperatures corresponding to predicted respiration rates of 40, 60, and 80 breaths/min.

Sex	Growth Stage	RR Criterion (Breaths/min)	Model-Derived Reference Temperature (°C)	95% CI
Female	Adult	40	22.14	21.46–22.71
Female	Adult	60	26.73	26.12–27.40
Female	Adult	80	29.99	29.07–31.11
Male	Adult	40	21.20	19.99–22.20
Male	Adult	60	25.79	24.75–26.84
Male	Adult	80	29.05	27.92–30.34
Female	Growing	40	23.12	22.41–23.89
Female	Growing	60	27.71	26.87–28.68
Female	Growing	80	30.97	29.83–32.41
Male	Growing	40	22.99	22.33–23.65
Male	Growing	60	27.59	26.85–28.44
Male	Growing	80	30.85	29.77–32.20

## Data Availability

The data were not deposited in an official repository. The data supporting the findings are available upon request from the corresponding author.
